# East London Modified-Broset as Decision-Making Tool to Predict Seclusion in Psychiatric Intensive Care Units

**DOI:** 10.3389/fpsyt.2017.00194

**Published:** 2017-10-04

**Authors:** Felice Loi, Karl Marlowe

**Affiliations:** ^1^Juniper Court Churchill Hospital CAS Behavioural Health, London, United Kingdom; ^2^Millharbour PICU Mile End Hospital East London NHS Foundation Trust, London, United Kingdom; ^3^Centre for Psychiatry Queen Mary University of London, London, United Kingdom

**Keywords:** behavior, coercion, decision-making, prediction, seclusion, violence

## Abstract

Seclusion is a last resort intervention for management of aggressive behavior in psychiatric settings. There is no current objective and practical decision-making instrument for seclusion use on psychiatric wards. Our aim was to test the predictive and discriminatory characteristics of the East London Modified-Broset (ELMB), to delineate its decision-making profile for seclusion of adult psychiatric patients, and second to benchmark it against the psychometric properties of the Broset Violence Checklist (BVC). ELMB, an 8-item modified version of the 6-item BVC, was retrospectively employed to evaluate the seclusion decision-making process on two Psychiatric Intensive Care Units (patients *n* = 201; incidents *n* = 2,187). Data analyses were carried out using multivariate regression and Receiver Operating Characteristic (ROC) curves. Predictors of seclusion were: physical violence toward staff/patients OR = 24.2; non-compliance with PRN (pro re nata) medications OR = 9.8; and damage to hospital property OR = 2.9. ROC analyses indicated that ELMB was significantly more accurate that BVC, with higher sensitivity, specificity, and positive likelihood ratio. Results were similar across gender. The ELMB is a sensitive and specific instrument that can be used to guide the decision-making process when implementing seclusion.

## Introduction

Reducing restrictive practice *via* the reduction of seclusion has become one of the top priorities of many health authorities (1–3). Although rates of seclusion have fallen worldwide (4, 5), rates of violence on psychiatric wards have not substantially changed with evidence denoting an upward trend (6, 7), arguably indicating that seclusion is a therapeutic option increasingly reserved to contain more extreme cases of violence. The use of seclusion is controversial (8, 9). Generally implemented as a last resort treatment (10), at times alone, more often in combination with other, mainly pharmacological approaches (11, 12), seclusion use is criticized as an unjustifiable and potentially traumatic practice for patients (13–17). These claims, however, remain hotly debated (18). Seclusion, in fact, can be an effective and safe therapeutic tool in selected cases (19, 20) with limited evidence of harmful effects to patients (21–23). A lack of appropriate psychometric tools that standardize an objective seclusion approach to violent patients based on degree of risks is identified as one of the major flaws by its detractors.

In spite of the fact that assessment of risk of violence on psychiatric wards has become much easier and more reliable thanks to the implementation of validated instruments like the Broset Violence Checklist (BVC) (24), or the Staff Observation Aggression Scale Revised (25), the decision to seclude a patient remains mostly an *ad hoc* process. This is not surprising considering the dynamic nature of a decision-making process (26) that reflects a complex interplay of multiple patient-related (5, 27–29), environmental-related (9, 30–32), and team-related factors (33–35).

Indeed, assessment tools that may predict seclusion as part of a therapeutic care plan have become available (36, 37), but they are research-oriented, and far too impractical to be used in every day clinical practice, as they depend on prior collection of detailed risk information regarding demographic, historical, and behavioral factors. The complexity and length of these tools means they cannot meet the requirements of a quick decision-making process by the frontline staff on the ward, particularly needed when dealing with rapidly unfolding situations (38).

Based on our clinical experience of Psychiatric Intensive Care Units (PICUs), and supported by evidence from the literature on seclusion, we extended on our previous exploratory work (39) on the development of a modified version of the BVC, the East London Modified-Broset (ELMB), taking into consideration two factors affecting staff’s seclusion decision-making when containing high acuity behaviors, that is, patient’s degree of compliance with PRN (pro re nata) medications, *per os* (P.O.) or intramuscular (I.M.), and response to de-escalation (verbal and/or physical) or lack thereof.

In this case-control study, we retrospectively employed ELMB in a sample of patients consecutively admitted to two PICUs over a 3-year period. The aims of this study were to analyze the predictive characteristics of ELMB with respect to secluding adult psychiatric inpatients at high risk of violence; to describe ELMB’s accuracy levels; its ability to discriminate patients’ degrees of risk; to delineate ELMB as a seclusion decision-making tool. In the absence of a gold-standard seclusion tool, the BVC was used as a benchmark instrument to compare the discriminatory characteristics of ELMB.

## Materials and Methods

### Participants

The sample consisted of 252 inpatients (males *n* = 130; females *n* = 122) consecutively admitted to Millharbour (male) and Rosebank (female) PICUs at Tower Hamlets Centre for Mental Health, Mile End Hospital (London, UK), between June 2012 and May 2015. The two PICUs serve the London Borough of Tower Hamlet catchment area with an estimated population of about 280,000, whereas Rosebank PICU covers two additional London boroughs, with an aggregated overall population of over 550,000. The male PICU, with a bed capacity of 14, and the female PICU of 11, serve primarily Mile End Hospital in Tower Hamlets with an overall capacity of 76 general adult beds. Patients were included in the study if they had a confirmed or suspected diagnosis of psychiatric disorder meeting the criteria of DSM-IV-TR (40), and were 18–65 years old. Two of the individual participants in the female sample were older than 65 (female age *range* = 18–69; male age *range* = 18–53). Although individual participants with age >65 would usually be treated on an Old Age Psychiatry ward, in selected circumstances, these patients would be admitted to the general adult PICU based on severity of violence and physical fitness. Patients with behavioral disturbance secondary to general medical condition were excluded. Only one individual diagnosed with Limbic Encephalitis with *N*-methyl-d-aspartate receptor antibodies was thus excluded from the analyses. Because of the “revolving door nature” of patients admitted to PICUs (41), in order to minimize confounders on the seclusion decision-making process of patients already known to each PICU (42), only the first admission to PICU of patients’ multiple admissions were included for ratings. Finally, 51 individual participants (20%) were excluded from the analyses due to inadequate information reported in the clinical records.

### Psychiatric Assessment

A specialist in General Adult Psychiatry (Felice Loi) was responsible for the retrospective ratings of the participants’ clinical entries in the electronic medical records. For the purpose of this study, an incident was defined as any clinical event that led to an interaction between a patient and one or more members of the clinical team and which involved the use of de-escalation techniques (verbal and/or physical), and/or the administration of PRN sedative medications (P.O. and/or I.M.—see below).

### Psychometric Tool Characteristics and Scoring System

**Figure 1:**
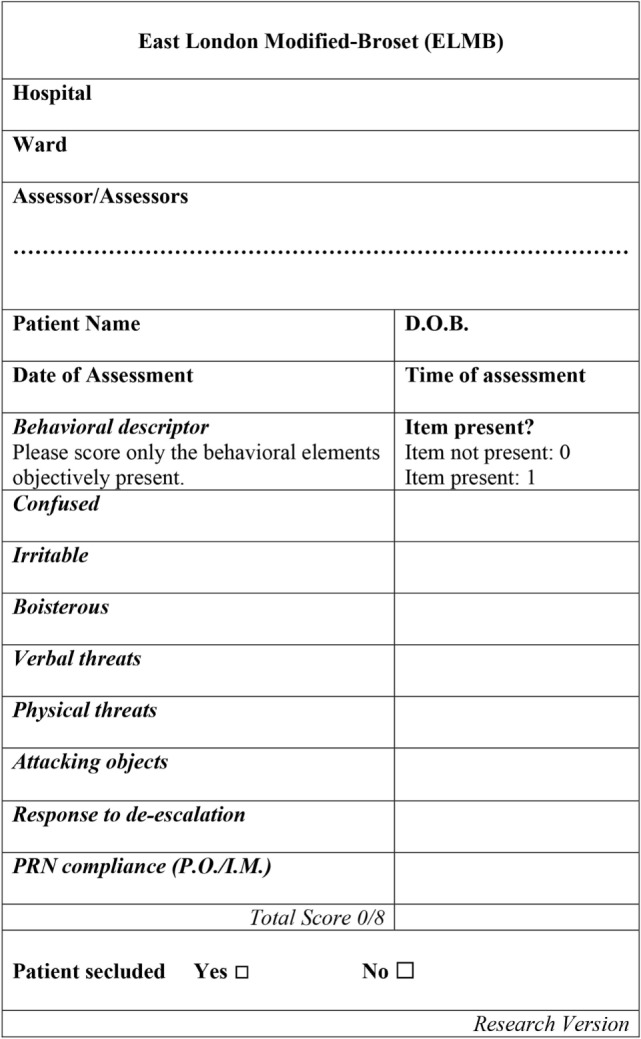
The East London Modified-Broset (ELMB) assessment tool. Note. D.O.B., patient’s date of birth. P.O., *per os*. I.M., intramuscular medication. See text for behavioral items description.

East London Modified-Broset (Figure 1) is an 8-item checklist based on the 6-item BVC, of which follows similar compilation rules (24), albeit not for all items. In our setting, a patient was deemed “*confused*” as synonym for severely thought-disordered, where the level of thought disorder interfered considerably with the ability to process information provided during interaction with staff (for instance, significant thought blocking, illogicality, racing thoughts with overinclusive thinking, and loosening of associations) which could be associated or not with cognitive disorientation to time, place, or person. The items “*irritable*” (significant affect reactivity disproportionate to the level of stimuli available); “*boisterous*” (being loud, banging on items in order to attract someone’s attention without intention to damage hospital property); “*verbal threats*” (articulating verbal insults with or without menacing words which threaten the victim’s perceived sense of safety); “*physical threats*” (threatened or actual violence); and “*attacking objects*” (hitting objects with the intention to cause damage to property), were rated as in the original BVC, that is, 0 if the behavior was not present, 1 if the behavior was present. The main difference between the two tools is the inclusion of two additional items in the ELMB: (1) “*response to de-escalation*” and (2) “*PRN compliance*” (i.e., whether the patient has accepted or not any PRN medications offered). The item “*response to de-escalation*” deserves further specification. A patient whose physically aggressive behavior led to members of staff employing measures of physical control over his behavior (i.e., any type of physical contact between a member of staff and a patient) was generally accompanied by measures of verbal de-escalation which could, within a variable length of time, lead to the patient regaining full control over his own behavior with resolution of the behavioral crisis. In this case, the patient was deemed to have responded to de-escalation and his behavior rated on the ELMB as “0” as opposed to the inability to respond to staff’s interventions and the consequent escalation of behavior, an outcome that would be rated as “1”. The number of positive items was added up to reach a total possible score of 8/8 on the ELMB, and of 6/6 on the BVC with higher scores indicating higher levels of behavioral disturbance (implicit for the ELMB, of reduced ability to cooperate with staff). The inclusion of these two factors in the ELMB is supported by evidence indicating that staff’s interaction with patients, represents the most important trigger to violence in almost 40% of cases (43) often with patients perceiving some form of imposed restriction. Overall, the BVC is a risk prediction tool of violence occurring within 24 h following an assessment, while the ELMB is a decision-making tool to be used at the time an incident is unfolding.

### Data Analysis

Statistical analyses were carried out using Stata SE 14.1 for Mac (44) and MedCalc Statistical Software for Windows version 16.2 (45). Sample size required was calculated with G*Power Version 3.1.9.2 for Mac (46). A minimum sample size of 158 individual participants was required to achieve a pre-specified power of 85% for a one-tailed probability. To minimize bias of the logistic regression model (47), a minimum number of 10 events per variable was calculated by multiplying the number of predictors (8) by a constant K (10 seclusion events), thus yielding a minimum total number of 80 seclusion events.

Parametric and non-parametric analyses along with effect size (ES) were calculated to identify differences in demographic and clinical variables among secluded and non-secluded patients. In order to improve accuracy of estimates, *bootstrap* test statistics were implemented with bias corrected accelerated (BCa) computation of confidence intervals. Prior to running bootstrap analyses, a seed number was set in order to ensure reproducibility of the findings (48) generating a random numeric sequence taken from a pound sterling note (set seed number 78484608). Number of bootstrap repetitions was set to 1,000. Analyses were carried out for the whole sample and, separately, for the male and female individual participants to detect a main effect of gender.

Model accuracy for each of the two tools was estimated using Receiver Operating Characteristics (ROC) curves with overall performance expressed by the area under the curve (AUC) index. The Youden Index (YI) was calculated in order to identify the optimum threshold point on the ROC curve correspondent to best compromise between true positive and false positive rates (49). Pairwise difference of the AUCs for the two tools was calculated and ES of the differences reported. In addition, positive (LHR^+^) and negative likelihood (LHR^−^) ratios were reported to quantitatively reflect the accuracy of the tools to confirm the presence of a true positivity or a true negativity compared to participants that, respectively, needed to be secluded or that did not need to be secluded.

### Model Building

In order to identify ELMB psychopathological items that were predictive of the use of seclusion, logistic regression analyses were carried out. Chi-square analyses were initially performed to identify psychopathological items that were significantly associated with the use of seclusion. All eight ELMB predictors achieved significance level. Therefore, they were forced-entered into a binary regression model (BRM) as independent variables, with seclusion as dependent variable. In preliminary analyses, “*response to de-escalation*” disclosed very large odds ratios (ORs) = 451, SE = 327.2, *p* = 0.000, 95% CI (108.8, 1,869.8) suggesting a problem of separation (50). In conditions of separation, one or more predictors exert a very high predictive effect, which leads to instability in the BRM whose maximum likelihood estimates cannot be calculated. In such conditions, a penalized likelihood estimate can be more appropriately calculated with Firthlogit method (51) implemented in Stata with firthlogit command (52). However, since Firthlogit algorithm did not appreciably correct the bias, albeit not desirable due its strong predictive effect (53), “*response to de-escalation*” was constrained in the model (i.e., retained to exert its overall effect but without estimating its predictive value). Using a hierarchical method, two constrained models were thus developed based on prior findings from seclusion literature, our own clinical experience, and statistical measures of fit, including Aikake’s Information Criterion (AIC). Both models incorporated *physical threats, attacking objects*, and *PRN compliance*, with the difference of *verbal threats*, which was present only in model 1. This is because verbal threatening behavior can shift staff’s decision to seclude a patient based on findings from previous studies (27, 54, 55). Both models achieved high significance levels. However, although model 1 performed slightly better in terms of AIC (411.763 vs. 419.321), regression diagnostics disclosed concerns due to *verbal threats’* uncentered VIFs > 2.30, which led us to choose model 2.

## Results

### Demographic and Clinical Variables

Table 1 shows the sample’s characteristics clustered by seclusion status. There was no statistically significant difference in age, *t*_(199)_ 1.23, *p* = 0.217; gender, χ^2^ (3, *N* = 201) = 0.976, *p* = 0.32; ethnicity, χ^2^ (1, *N* = 201) = 0.211, *p* = 0.65; type of primary diagnosis, two-sided *Fisher’s Exact*: *p* = 0.771; antisocial, χ^2^ (1, *N* = 201) = 0.0004, *p* = 0.98; and borderline characteristics, χ^2^ (1, *N* = 201) = 0.285, *p* = 0.59; nor substance abuse in the 2 weeks prior to admission, χ^2^ (1, *N* = 194) = 0.356, *p* = 0.55. There was no statistically significant difference among male and female individuals in number of seclusion and non-seclusion incidents, χ^2^ (1, *N* = 2,187) = 0.135, *p* = 0.71.

**Table 1:** Demographic and clinical data.

Descriptor	Non-Secluded	Secluded	*p* < 0.05
	*n* (%)	*n* (%)	
**Participants (*n* = 252)**			
Included (*n* = 201)	143 (71.1)	58 (28.9)	
Age, years (M, SD)	33.9 (11)	31.5 (10.9)	0.21
Sex			0.32
Male	63 (31.3)	30 (14.9)	
Female	80 (39.8)	28 (13.9)	
Incidents			0.71
Male	796 (36.4)	71 (3.25)	
Female	1,206 (55.14)	114 (5.21)	
Ethnicity			0.65
Caucasian	35 (17.4)	16 (8)	
Non-Caucasian	108 (53.7)	42 (20.9)	
Diagnosis spectra			0.77
Schizophrenia	49 (24.4)	20 (10)	
Affective	37 (18.4)	19 (9.5)	
Schizoaffective	43 (21.4)	13 (6.5)	
Personality disorder	12 (6)	5 (2.5)	
Substance Abuse	2 (1)	1 (0.5)	
Antisocial traits			0.98
Present	52 (25.9)	21 (10.5)	
Non-present	91 (45.3)	37 (18.4)	
Borderline traits			0.59
Present	32 (15.9)	11 (5.5)	
Non-present	111 (55.2)	47 (23.4)	
Substance abuse^a^ (*n* = 194)			0.55
Present	57 (29.4)	27 (13.9)	
Non-present	79 (40.7)	31 (16)	
History of seclusion (*n* = 168)	123 (73.2)	45 (26.8)	0.018
Present	36 (21.4)	22 (13.1)^b^	
Non-present	87 (51.8)	23 (13.7)	

*^a^Substance abuse in the 2 weeks prior to admission to hospital or Psychiatric Intensive Care Units*.

*^b^Standardized residual = +1.6 at p < 0.05 level. See text for Chi-square values*.

In 33 (16.4%) out of 201 individual participants, medical records information regarding past history of seclusion was missing. Chi-square analyses identified that individuals who were secluded were associated with a prior history of seclusion, χ^2^ (1, *N* = 168) = 5.61, *p* = 0.018, *Cramer’s V*: 0.17. Bootstrap binary logistic regression confirmed that having a history of seclusion had a positive predictive value on the odds of being secluded during a subsequent admission OR = 2.31, *p* = 0.023, BCa 95% CI (1.12, 4.76). However, the effect size of the model explained only 2.8% of the variance: *pseudo R*^2^ = 0.028, indicating minimal influence of the overall odds, and the potential confounder of the results.

In the sample of patients excluded from the analyses, a statistically significant difference was found among secluded and non-secluded in type of primary diagnosis only, two-sided *Fisher’s Exact*: *p* = 0.001, with most of individuals diagnosed with paranoid schizophrenia (*n* = 19 or 59.4%).

### Psychometric Tools

#### East London Modified-Broset

Of the 2,187 valid incidents for the participants regardless of gender, 185 led to seclusion (M = 6.21, SD = 1.2), while 2,002 did not (M = 3.41, SD = 1.9). This difference on the ELMB scores [M = −2.79, SE = 0.91, 95% CI (−2.97, −2.6)], was highly significant: [*t*_(2,185)_ −30.47, SE = 1.52, *p* = 0.0000, BCa 95% CI (−33.4, −27.5)], with large ES: Cohen’s *d* = 2.3, 95% CI (2.1, 2.5). Mean difference analyses were repeated for both the male and female groups separately to identify an effect of gender. For the female group, a total of 1,320 incidents were considered, of which 1,206 (M = 3.44, SD = 1.18) did not lead to the use of seclusion, while in 114 cases, individuals were secluded (M = 6.32, SD = 1.08). This difference on ELMB scores [M = −2.88, SE = 0.11, 95% CI (−3.1, −2.7)], was highly significant [*t*_(1,318)_ −25.11, SE = 1.48, *p* = 0.0000, BCa 95% CI (−28, −22.2)], with large ES: Cohen’s *d* = 2.46, 95% CI (2.24, 2.67). Among the male participants, results were essentially the same: total number of incidents 867, of which 796 (M = 3.37, SD = 1.21) did not lead to seclusion while 71 (M = 6.02, SD = 1.38) were seclusion events. This difference on ELMB scores [M = −2.66, SE = 0.15, 95% CI (−2.96, −2.36)] was highly significant [*t*_(865)_ −17.49, SE = 1.64, *p* = 0.0000, BCa 95% CI (−20.7, −14.3)], with large ES: Cohen’s *d* = 2.16, 95% CI (1.9, 2.42).

#### Broset Violence Checklist

Results of *bootstrap t*-tests analyses were essentially similar. There were 2,187 valid incidents for the total sample, of which 185 led to seclusion (M = 4.74, SD = 1), while 2,002 did not (M = 3.25, SD = 1.1). This difference on scores [M = −1.48, SE = 0.08, 95% CI (−1.64, −1.31)], was highly significant: [*t*_(2,185)_ −17.6, SE = 1.11, *p* = 0.000, BCa 95% CI (−19.7, −15.4)], with large ES: Cohen’s *d* = 1.35, 95% CI (1.51, 1.2). In subset analyses for the female group, a total of 1,320 incidents were considered, of which 1,206 (M = 3.27, SD = 1.1) did not lead to the use of seclusion, while in 114 cases, individual participants were secluded (M = 4.83, SD = 0.88). This difference on scores [M = −1.56, SE = 0.1, 95% CI (−1.8, −1.36)], was highly significant [*t*_(1,318)_ −14.97, SE = 1.08, *p* = 0.000, BCa 95% CI (−17.1, −12.8)], with large ES: Cohen’s *d* = 1.5, 95% CI (1.7, 1.3). Among the male participants, there were 867 events, of which 796 did not lead to seclusion (M = 3.24, SD = 1.14), and 71 (M = 4.6, SD = 1.15) were seclusion incidents. This difference on scores [M = −1.35, SE = 0.14, 95% CI (−1.62, −1.07)] was highly significant [*t*_(865)_ −9.56, SE = 1.2, *p* = 0.000, BCa 95% CI (−11.9, −7.23)], with large ES, Cohen’s *d* = 1.24, 95% CI (1.5, 0.9).

Analyses were repeated to compare male and female patients on levels of behavioral disturbance as measured by ELMB and the BVC for all seclusion and non-seclusion incidents. No statistically significant difference was found (results not reported).

#### Regression Model

The results of Firthlogit (constrained) model for the whole sample of participants, as well as the female and male groups are reported in Tables 2–4, respectively. The analyses show that the most important predictors of seclusion were acts of *physical threats* (males OR = 18.9; females OR = 33.1; combined OR = 24.2), *attacking objects* (males OR = 2; females OR = 3.6; combined OR = 2.9), *PRN compliance* (males OR = 18.4; females OR = 7.5; combined OR = 9.85).

**Table 2:** Firthlogit regression (constrained model): coefficients and ORs of the model predicting whether a participant, regardless of gender, was secluded.

Factors	Coef.^a^	SE^b^	OR^c^	*Z*	*p* > |z|	95%^d^ CI OR	
Seclusion						Lower	Upper
Physical threats	3.18	0.36	24.2	8.85	0.000	11.9	48.9
Attacking objects	1.07	0.2	2.91	5.35	0.010	2	4.3
PRN compliance^e^	2.29	0.2	9.85	11.6	0.001	6.7	14.5
Response to de-escalation	Omitted^f^						
Constant	−5.47	0.36	0.004	−15.6	0.000	0.002	0.008

*^a^Coefficient*.

*^b^SE of the coefficient*.

*^c^OR, odds ratio*.

*^d^Confidence interval OR*.

*^e^PRN, pro re nata medications*.

*^f^Response to de-escalation (unconstrained) Coef. = 5.9 OR = 362.02 (SE = 234.7), *z* = 9.09, *p* = 0.000, 95% CI (101.6, 1,289.9)*.

*Penalized log likelihood = −404.25, likelihood-ratio χ^2^ (1, N = 2,187) = 399.17, p = 0.0000*.

**Table 3:** Firthlogit regression (constrained model): coefficients and ORs of the model predicting whether a female participant was secluded.

Factors	Coef.^a^	SE^b^	OR^c^	*Z*	*p* > |z|	95%^d^ CI OR	
Seclusion						Lower	Upper
Physical threats	3.49	0.55	33.1	6.33	0.000	11.2	97.7
Attacking objects	1.28	0.24	3.6	5.24	0.000	2.2	5.9
PRN compliance^e^	2.01	0.24	7.5	8.29	0.000	4.6	12.1
Response to de-escalation	Omitted^f^						
Constant	−5.87	0.55	0.003	−10.6	0.000	0.0009	0.008

*^a^Coefficient*.

*^b^SE of the coefficient*.

*^c^OR, odds ratio*.

*^d^Confidence interval OR*.

*^e^PRN, pro re nata medications*.

*^f^Response to de-escalation (unconstrained) Coef. = 5.9, OR = 372.8, SE = 312.4, *z* = 7.07, *p* = 0.000, 95% CI (72.1, 1,926.7)*.

*Penalized log likelihood = −247.82, likelihood-ratio χ^2^ (1, N = 1,320) = 243.05, p = 0.0000*.

**Table 4:** Firthlogit regression (constrained model): coefficients and ORs of the model predicting whether a male participant was secluded.

Factors	Coef.^a^	SE^b^	OR^c^	*Z*	*p* > |z|	95%^d^ CI OR	
Seclusion						Lower	Upper
Physical threats	2.94	0.46	18.9	6.33	0.000	7.6	47.1
Attacking objects	0.71	0.35	2	2.03	0.043	1.02	4.01
PRN compliance^e^	2.91	0.36	18.4	8.15	0.000	9.12	37
Response to de-escalation	Omitted^f^						
Constant	−5.06	0.45	0.006	−11.1	0.000	0.002	0.015

*^a^Coefficient*.

*^b^SE of the coefficient*.

*^c^OR, odds ratio*.

*^d^Confidence interval OR*.

*^e^PRN, pro re nata medications*.

*^f^Response to de-escalation (unconstrained) Coef. = 5.4, OR = 232.9, SE = 196.7, *z* = 6.45, *p* = 0.000, 95% CI (44.5, 1,219.1)*.

*Penalized log likelihood = −146.56, likelihood-ratio χ^2^ (1, *N* = 867) = 151.73, *p* = 0.0000*.

Although only three of the eight behavioral items (*physical threats, attacking objects, PRN compliance*) listed in the ELMB achieved significance level, all predictors were included in the final ROC analyses as these factors were judged to aid team discussion around the appropriateness of using seclusion. This choice was based on the clinical ward experience that the interaction between a member of staff and a patient is not motivated only by the occurrence of a sudden episode of physical aggression, as most incidents are dealt with prior to an escalation to an aggressive episode. The early identification of behavioral elements such as significant thought disorder, irritability, boisterousness, verbal threats, associated or not with aggression toward items, offer an immediate opportunity for early therapeutic engagement (through the offer of de-escalation approaches and/or PRN mediations), which prevents, in many cases, the occurrence of incidents of actual violence. In support of this approach, the bootstrap logistic regression analyses indicated that higher scores on ELMB were more positively predictive of the use of seclusion (Table 5) female OR = 7.2; male OR = 4.5; combined OR = 5.7, with similar trends, albeit with lower ES of the model, described by BVC scores (bottom half of Table 5). In order to identify which scores on the ELMB were associated with the highest predictive value of seclusion, incidents were stratified into two clusters, one with ELMB scores ≤4 and one with ELMB scores >4 based on *Youden index* associated criterion >4. Bootstrap analyses (Table 6) showed that ELMB scores in the former cluster of incidents (*n* = 1689) were not predictive of seclusion OR = 1.5, *p* = 0.37, BCa 95% CI (0.63, 3.44), while incidents with ELMB scores >4 (*n* = 498) were significantly predictive of seclusion OR = 5.9, *p* < 0.000, BCa 95% CI (4.33, 8.11), pseudo *R*^2^ = 0.29 (29%).

**Table 5:** Bootstrap logistic regression: coefficients and ORs of the model predicting whether individual participants were secluded at higher East London Modified-Broset (ELMB) and Broset Violence Checklist (BVC) scores.

Factors	Sample	*n*	pR^2a^	Coef.^b^	SE^c^	OR^d^	*Z*	*p* > |z|	95%^e^ CI OR	

Seclusion									Lower	Upper
ELMB	M + F	2,187	0.51	1.74	0.114	5.7	16.3	0.000	4.6	7.1
ELMB	F	1,320	0.56	1.97	0.156	7.2	12.7	0.000	5.3	9.7
ELMB	M	867	0.45	1.49	0.167	4.5	8.93	0.000	3.2	6.2
BVC	M + F	2,187	0.23	1.30	0.092	3.7	13.1	0.000	3	4.5
BVC	F	1,320	0.27	1.52	0.127	4.6	11.6	0.000	3.5	5.9
BVC	M	867	0.18	1.05	0.154	2.9	7.12	0.000	2.1	3.8

*Each model is run separately. M, male sample; F, female sample*.

*^a^Pseudo R^2^*.

*^b^Coefficient*.

*^c^SE of the coefficient*.

*^d^OR, odds ratio*.

*^e^Confidence interval OR*.

**Table 6:** Bootstrap logistic regression: ORs of the model to identify specific East London Modified-Broset (ELMB) and Broset Violence Checklist threshold scores predictive of seclusion.

Factors seclusion	Sample type	*n*	pR^a^	SE^b^	OR^c^	*p* > |z|	95%^d^ CI OR	
							Lower	Upper
ELMB ≤ 4	M + F	1,689	0.008	0.43	1.5	0.37	0.6	3.4
ELMB > 4	M + F	498	0.29	0.16	5.9	0.000	4.3	8.1
ELMB ≤ 4	F	1,008	0.02	0.64	2	0.24	0.6	6.5
ELMB > 4	F	312	0.30	0.2	6.1	0.0000	4.1	9
ELMB ≤ 4	M	681	0.006	0.47	1.3	0.51	0.5	3.4
ELMB > 4	M	186	0.28	0.72	5.7	0.000	3.2	9.9

*Each model is run separately. M, male sample; F, female sample*.

*^a^Pseudo R^2^*.

*^b^SE of the coefficient*.

*^c^OR, odds ratio*.

*^d^Confidence interval OR*.

#### ROC Analyses

**Figure 2:**
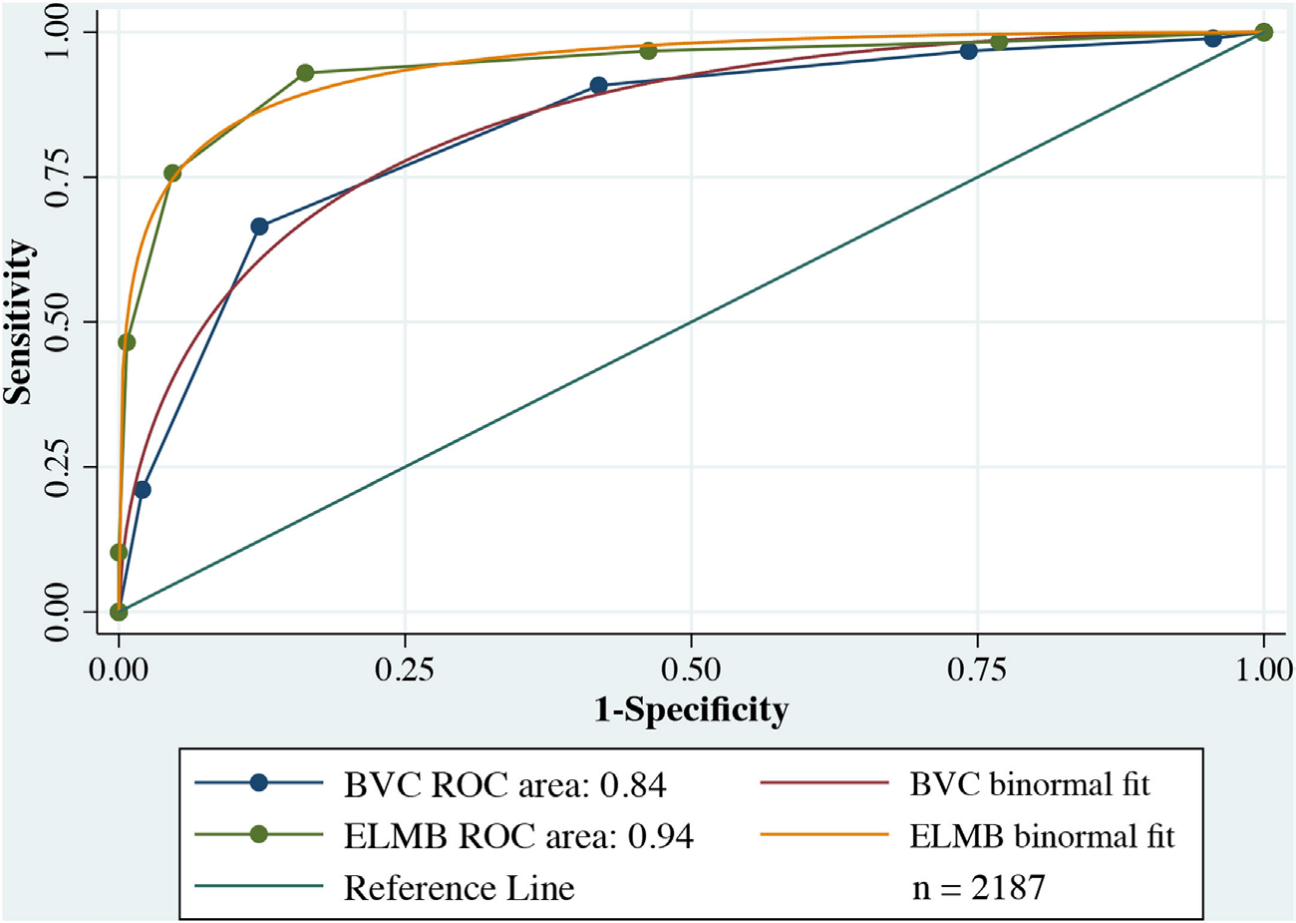
Receiver operating characteristic (ROC) comparison plots Broset Violence Checklist (BVC) vs. East London Modified-Broset (ELMB).

Figure 2 plots a comparison of ROC curves for the whole sample for ELMB and BVC. The difference between the two AUCs (Table 7) was highly significant, Δ AUCs = 0.10, SE = 0.0079, *p* < 0.0001, BCa 95% CI (0.085, 0.117), with large ES: *z* = 12.6 indicating that ELMB’s overall discriminatory characteristics outperformed the BVC by a large margin. The Youden Index and the associated criterion for the BVC and ELMB are reported in Table 7. The best trade-off between true positive and false positive rates as identified by the Youden Index lies at a score >4 for both tools. Table 7 also shows that at the Youden Index associated criterion >4, the sensitivity and specificity are higher for ELMB as compared to the BVC. Accuracy measures for the whole sample are reported in Table 8. From a close examination of Table 8, two clear trends emerge: (1) at *Youden Index* associated criterion >4 and (2) with ELMB score >6 (respectively indicating patients who achieved an ELMB score >4 and those with a score above 6). In the first case, the classification system indicated that ELMB was superior to the BVC (similar *LHR^+^* but *LHR^−^* and *NPV* significantly greater for ELMB) across all of the parameters, but for *specificity* (BVC = 87.7% > ELMB = 83.7%) and *FPR* (BVC = 12% < ELMB = 16%). At cut-off > 6, ELMB significantly outperformed BVC with an extremely high *specificity* (99.3%), low *FPR* (0.7%), and high *PPV* (96.4%), and *LHR^+^* significantly higher (66.5) than what normally expected (≥20) of tests’ requirements (56). Results were similar for the female and male groups (Tables 9 and 10).

**Table 7:** Pairwise comparison of receiver operating characteristic curves: *n* = 2,187 incidents.

	Tool	

Broset violence checklist		East London Modified-Broset
	Statistic	
0.84	AUC	0.94
0.0151	SE	0.0092
22.07	*Z*	40.92
<0.0001	*p*	<0.0001
0.81–0.87	AUC 95% CI	0.92–0.96
0.54	YI	0.77
0.48–0.61	YI 95% CI	0.72–0.80
>4	Criterion	>4
66.49	Sens.	92.97
87.71	Spec.	83.72

*AUC, area under the curve; Sens., sensitivity; Spec., specificity at YI, Youden index level*.

**Table 8:** Coordinates of the receiver operating characteristic curves for the whole sample (*n* = 2,187 incidents).

Tool	Cut-off	Sens.	Spec.	FPR	(LHR^+^)	(LHR^−^)	PPV^a^	NPV^a^
BVC	>0	100	0.05	0.95	1.00	0.00	28.9	100
	>1	98.9	4.45	0.955	1.04	0.24	29.6	91.0
	>2	96.8	25.8	0.74	1.30	0.13	34.6	95.1
	>3	90.8	58.1	0.42	2.17	0.16	46.8	94.0
	>4^b^	66.5	87.7	0.12	5.41	0.38	68.7	86.6
	>5	21.1	97.9	0.02	10.3	0.81	80.7	75.3
	>6	0.00	100	0.0	–	1.00	–	71.1
ELMB	>0	100.0	0.1	0.9	1.00	0.00	28.9	100.0
	>1	100.0	3.95	0.6	1.04	0.00	29.7	100.0
	>2	98.4	23.1	0.77	1.3	0.07	34.2	97.2
	>3	96.8	53.7	0.46	2.1	0.06	46.0	97.6
	>4^b^	92.9	83.7	0.16	5.7	0.08	69.9	96.7
	>5	75.7	95.3	0.047	16.1	0.26	86.8	90.6
	**>6**	**46.5**	**99.3**	**0.007**	**66.5**	**0.54**	**96.4**	**82.0**
	>7	10.3	100.0	0.0	–	0.9	100.0	73.3
	>8	0.00	100.0	0.0	–	1.00	–	71.1

*Bold fonts indicate East London Modified-Broset (ELMB) patients’ average score and relative measures of accuracy at threshold chosen by ward staff*.

*FPR, false prediction rate; (LHR^+^), positive likelihood ratio; (LHR^−^), negative likelihood ratio; PPV, positive prediction rate; NPV, negative prediction rate; BVC, Broset Violence Checklist*.

*^a^Calculated at prevalence rate of 28.9%*.

*^b^Indicates best trade-off. Cut-off positive if ≥0*.

**Table 9:** Coordinates of the receiver operating characteristic curves for the female sample (*n* = 1,320 incidents).

Tool	Cut-off	Sens.	Spec.	FPR	(LHR^+^)	(LHR^−^)	PPV^a^	NPV^a^
BVC	>1	100.00	3.73	0.63	1.04	0.00	14.4	100.0
	>2	99.12	25.70	0.74	1.33	0.034	17.7	99.5
	>3	92.98	57.30	0.43	2.18	0.12	26.0	98.1
	>4^b^	68.42	87.6	0.12	5.50	0.36	47.0	94.5
	>5	22.81	98.6	0.01	16.18	0.78	72.3	88.8
	>6	0.00	100.0	0	–	1.00	–	86.1
ELMB	>0	100.00	0.08	0.99	1.00	0.00	13.9	100.0
	>1	100.00	3.23	0.68	1.03	0.00	14.3	100.0
	>2	99.12	22.8	0.77	1.28	0.038	17.2	99.4
	>3	99.12	52.2	0.48	2.08	0.017	25.1	99.7
	>4^b^	96.49	83.2	0.17	5.76	0.042	48.2	99.3
	>5	77.19	95.4	0.05	16.93	0.24	73.2	96.3
	**>6**	**48.25**	**99.4**	**0.006**	**83.12**	**0.52**	**93.1**	**92.2**
	>7	11.40	100.0	0	–	0.89	100.0	87.5
	>8	0.00	100.0	0	–	1.00	–	86.1

*Bold fonts indicate East London Modified-Broset (ELMB) patients’ average score and relative measures of accuracy at threshold chosen by ward staff*.

*FPR, false prediction rate; (LHR^+^), positive likelihood ratio; (LHR^−^), negative likelihood ratio; PPV, positive prediction rate; NPV, negative prediction rate; BVC, Broset Violence Checklist*.

*^a^Calculated at prevalence rate of 13.9%*.

*^b^Indicates best trade-off. Cut-off positive if ≥0*.

**Table 10:** Coordinates of the receiver operating characteristic curves for the male sample (*n* = 867 incidents).

Tool	Cut-off	Sens.	Spec.	FPR	(LHR^+^)	(LHR^−^)	PPV^a^	NPV^a^
BVC	>0	100.00	0.13	0.99	1.00	0.00	14.9	100.0
	>1	97.18	5.53	0.95	1.03	0.51	15.3	91.8
	>2	92.96	25.9	0.74	1.25	0.27	18.0	95.5
	>3	87.32	59.3	0.41	2.15	0.21	27.3	96.4
	>4^b^	63.38	87.9	0.12	5.26	0.42	47.9	93.2
	>5	18.31	97	0.03	6.07	0.84	51.5	87.1
	>6	0.00	100.0	0	–	1.00	–	85.1
ELMB	>0	100.00	0.13	0.99	1.00	0.00	14.9	100.0
	>1	100.00	5.03	0.5	1.05	0.00	15.6	100.0
	>2	97.18	23.6	0.76	1.27	0.12	18.2	98.0
	>3	92.96	56.0	0.44	2.11	0.13	27.0	97.8
	>4^b^	87.32	84.4	0.16	5.61	0.15	49.5	97.4
	>5	73.24	95.1	0.05	14.95	0.28	72.4	95.3
	**>6**	**43.66**	**99.1**	**0.009**	**49.65**	**0.57**	**89.7**	**90.9**
	>7	8.45	100.0	0	–	0.92	100.0	86.2
	>8	0.00	100.0	0	–	1.00	–	85.1

*Bold fonts indicate East London Modified-Broset (ELMB) patients’ average score and relative measures of accuracy at threshold chosen by ward staff*.

*FPR, false prediction rate; (LHR^+^), positive likelihood ratio; (LHR^–^), negative likelihood ratio; PPV, positive prediction rate; NPV, negative prediction rate; BVC, Broset Violence Checklist*.

*^a^Calculated at prevalence rate of 14.9%*.

*^b^Indicates best trade-off. Cut-off positive if ≥0*.

## Discussion

The results of this study support ELMB as a promising seclusion decision-making and prediction tool for hic et nunc use on acute psychiatric wards. Three factors significantly predicted the occurrence of seclusion in a sample of acutely disturbed patients, namely *attacking objects, physical threats*, and *PRN compliance*. The effect size of these findings was moderate for the first factor, with a large effect for the second and the third factors. In addition, results were consistent across the male and female samples. This can be partly explained on one hand, by the involvement in emergency situations of composite teams with members responding from both PICUs (as well as from other acute wards within the hospital), who, therefore, share common approaches to violent behavior, on the other, by the characteristics common to patients generally admitted to these highly specialized wards. These results are in line with findings from previous studies (5, 27, 28, 55).

Lack of *response to de-escalation* was statistically significant and has been associated in previous studies with violent behavior leading to seclusion (54, 55). Mann-Poll et al. (57) found that patient’s uncooperativeness played a paramount role in the decision-making process over seclusion. In our study, however, the true magnitude of the effect of *response to de-escalation* on the final odds of being secluded could not be fully estimated due to issues of separation. Problems of separation in logistic regression are common in situations in which one or more factors in the model perfectly predict the outcome (58). We believe that perfect prediction was at play in this case, as the preliminary assumptions of the BRM were fully met. In addition, results were consistent across the male and female samples. This indicates that *response to de-escalation* had a disproportionate weight on the decision to seclude a patient and is largely independent of the combined effects of the other behavioral predictors (*attacking objects, physical threats, and PRN compliance*), because the inclusion in a non-constrained BRM of *response to de-escalation* alone did not significantly change the final odd ratios. This primacy of *response to de-escalation* over other factors in driving the decision-making process is a likely reflection of the strict compliance with the Mental Health Act Code of Practice in England (59) and the view that seclusion is a last-resort approach to violent behavior. This is also supported by the large discrepancy between ELMB’s identified optimal threshold to seclusion (Youden Index associated criterion > 4) and staff’s perception of patients’ risk levels appropriate to seclusion (ELMB>6). The items of *confused, irritable, boisterous, and verbal threats* did not predict the occurrence of seclusion, in line with our clinical experience that patients exhibiting these behaviors do not generally meet the threshold to being secluded.

Patients with history of seclusion were twice as likely to be secluded during subsequent admissions compared to never secluded patients, although the small to moderate effect size of the odds, indicated that a positive history of seclusion had a negligible influence on the decision to seclude an acutely unwell patient. Although this association has not so far been fully investigated, these results are broadly in line with those reported by other investigators. For instance, while Swett (60) described a non-significant trend, Thompson (61) indicated that patients with history of seclusion were more likely to be secluded during future re-admissions, between 43% and 81% of the cases. Georgieva et al. (55) also found that patients subjected to measures of coercion (seclusion and/or restraint) were 2.4 times more likely to have had a history of previous exposure to coercive measures. In the latter study, coerced patients were more frequently male, younger, more severely unwell, and uncooperative. It could be that, as proposed by Thompson (61), known violent patients might make staff less tolerant to aggressive behaviors, which may thus lead more easily to seclusion. However, Thompson did not employ measures of current violent behavior, which makes it difficult to draw a full comparison with the attitude of staff in the present study. Our findings showed that patients who were secluded scored significantly higher on ELMB (>6) than the best estimated threshold to seclusion (*Youden Index* associated criterion >4), indicating that staff tolerated very high levels of disturbed behavior before using seclusion to manage aggression. Unlike Thompson’s study, our sample included only the first admission to PICU of patients with multiple admissions, which minimized the chances that staff had secluded the same patient during a previous admission and thus reducing the influence of this confounder on the decision-making process. In addition, the effect of this confounder was minimized by the collective nature of the decision-making process that took place within a composite team of staff, several of whom generally did not have any prior knowledge of the patients’ circumstances at the time of the incident.

Substance abuse, common among psychiatric patients (62), and an important contributor to aggression (63–65), has been linked to violent behavior leading to seclusion (27, 66). In our study, no significant difference was found among secluded and non-secluded patients in substance abuse consumption. There was no other difference in demographic and other clinical characteristics among secluded and non-secluded individuals. These results are not surprising and echo the weak and variable associations between violence, use of seclusion, and demographic and clinical characteristics described in other studies (67, 68). However, these results could also be explained by the homogenous characteristics of patients admitted to PICUs. This arguably indicates that the decision to seclude a patient was mainly motivated by the degree of aggressive behavior regardless of other patients’ clinical or demographic background.

East London Modified-Broset and BVC scores were significantly higher among patients who were secluded as compared to non-secluded patients with large ES indicating that both tools were very good at discriminating degrees of aggressive behavior, with similar results in the male and female groups. Despite these similarities, we believe that ELMB has an important additional advantage over the BVC in that it allows a dynamic assessment of the patient’s response to staff’s interventions (last resort component), and allows staff to review behaviors in order to decide on initiating seclusion. Interestingly, these elements are also rehearsed in some of the major international psychiatric guidelines on the use of seclusion (10, 69, 70).

Receiver operating characteristic analyses indicated that the discriminatory power of ELMB was over 12 SDs higher than the BVC’s. The inclusion of patient’s response to staff’s pharmacological and behavioral interventions are the two key-factors setting it apart from the benchmarked BVC for the purpose of seclusion assessment. Regression models indicated that higher scores were more predictive of seclusion outcome, with almost a sixfold increase in the probability of being secluded with ELMB score > 6. The choice to consistently seclude a patient at a higher (>6) than the best identified threshold (Youden Index associated criterion > 4) speculatively delineates a minimum expected standard that is likely to have acted upon by staff to use seclusion as the very last resort, potentially at the cost of increased risks toward staff. Because of the important implications that the adoption of this course of action can have in terms of ward safety, it is not immediately apparent the rationale behind this decision. Beside the strict compliance with the Mental Health Act Code of Practice England (59), an additional reason might be found in the higher therapeutic cost (*FPR* = 16%) at the lower threshold > 4, which would have meant that up to 16 in 100 incidents could have been potentially misled seclusion events. Because of the inverse relationship between sensitivity and specificity, the choice of a lower threshold would have driven up sensitivity and *FPR* to the detriment of specificity (*FPR* = 1—*specificity*). The *FPR* is much higher at ELMB > 4 than that at a cut-off > 6 (*FPR* = 0.7%), where less than 1 incident in 100 was dealt with seclusion. This might have represented a far better cost-benefit option for the staff decision-making. It is the view of these authors, however, that an increase in the *FPR* would not automatically reflect an inappropriate increase of seclusion events. This is indicated by the ROC model, which identifies as a “true positive” any seclusion incident that has achieved a high score on the ELMB, compared to non-seclusion incidents that were scored low on the ELMB (i.e., “true negatives”). The seclusion of a patient with a low ELMB score (for instance, a patient secluded following an actual physical assault might score as low as 2 or 3) would be identified by the classification model as a false positive when it would indeed be a “true positive” (i.e., the patient needed to be secluded as immediate precaution for staff and patient safety).

An additional limitation lays in the retrospective nature of the study, which limits the generalizability of the findings. However, the sample size was generous and the results consistent across the male and female samples. Employing the BVC as standard of reference against the ELMB might be regarded as controversial given that the BVC was not designed as seclusion prediction tool. This limitation notwithstanding, the BVC displayed reasonable discriminatory properties non-dissimilar from that of other specific, albeit lengthy, seclusion instruments, like the Resident Assessment Instrument-Mental Health (71). Despite these limitations, our study presents several strengths. ELMB isolates well recognized behavioral components of violence and, unlike many other seclusion assessment tools, is short and focuses on how patients respond to staff’s interventions regardless of other demographic and clinical characteristics. As such, our tool is novel and has the potential to fill an important gap in seclusion clinical practice where it can standardize a consistent approach to the implementation of seclusion on the ward.

In the future, a prospective case-control study looking into the validation of ELMB as seclusion prediction and decision-making tool will be required in order to examine its discriminatory properties in real-time analysis. Finally, in a clinical panorama characterized by the absence of any seclusion discontinuation tools, it would be equally important to test ELMB in this aspect of the clinical assessment in light of the important repercussions that such an assessment can have on the safety of the ward and the patient’s care plan.

## Ethics Statement

This study was carried out in accordance with the recommendations of the Joint Research Management Office of Queen Mary Innovation Centre and East London NHS Foundation Trust who gave Ethical and R&D approval. Since the present research was limited to secondary use of information previously collected in the course of normal care (without an intention to use it for research at the time of collection), it was excluded from Research Ethics Committee (REC) review and approval. As such, the present research required only R&D approval by East London NHS Foundation Trust (ELFT). The study was sponsored by East London NHS Foundation Trust: protocol reference KO1308/1.

## Author Contributions

FL and KM conceptualized the work, interpreted the data, and wrote the manuscript. FL designed the research and acquired and analyzed the data. All authors approved the final version of the present manuscript to be published.

## Conflict of Interest Statement

The present research was conducted in the absence of any commercial or financial relationships that could be construed as a potential conflict of interest.
